# Inhibitory role of acyl homoserine lactones in hemolytic activity and viability of *Streptococcus pyogenes* M6 S165

**DOI:** 10.1038/srep44902

**Published:** 2017-03-17

**Authors:** Sunil D. Saroj, Linda Holmer, Júlia M. Berengueras, Ann-Beth Jonsson

**Affiliations:** 1 Department of Molecular Biosciences, The Wenner-Gren Institute, Stockholm University, 10691 Stockholm, Sweden

## Abstract

*Streptococcus pyogenes* an adapted human pathogen asymptomatically colonizes the nasopharynx, among other polymicrobial communities. However, information on the events leading to the colonization and expression of virulence markers subject to interspecies and host-bacteria interactions are limited. The interference of acyl homoserine lactones (AHLs) with the hemolytic activity and viability of *S. pyogenes* M6 S165 was examined. AHLs, with fatty acid side chains ≥12 carbon atoms, inhibited hemolytic activity by downregulating the expression of the *sag* operon involved in the production of streptolysin S. Inhibitory AHLs upregulated the expression of transcriptional regulator LuxR. Electrophoretic mobility shift assays revealed the interaction of LuxR with the region upstream of *sagA*. AHL-mediated bactericidal activity observed at higher concentrations (mM range) was an energy-dependent process, constrained by the requirement of glucose and iron. Ferrichrome transporter FtsABCD facilitated transport of AHLs across the streptococcal membrane. The study demonstrates a previously unreported role for AHLs in *S. pyogenes* virulence.

*S. pyogenes* is a common colonizer of the nasopharynx, a site inhabited by a plethora of other opportunistic Gram-positive and Gram-negative bacteria^1^,^2^. The bacterium is an adapted human pathogen, and colonization is usually asymptomatic. The bacterium can also readily colonize the skin; the other infrequent sites of colonization include the gastrointestinal tract and the lower female genital tract^3^. However, in certain cases, this bacterium is the causative agent of severe pharyngitis, streptococcal toxic shock syndrome, and necrotizing fasciitis^4^. Infections caused by *S. pyogenes* are epidemic and worldwide; the past decade has seen a steep increase in streptococcal infections^5^,^6^. The bacterium possesses the machinery to modulate virulence factors enabling adherence, invasion, and spread within the human host^7^.

In opportunistic pathogens, the expression of virulence factors is tightly regulated, a feat achieved by the phenomenon of quorum sensing^8^. The *S. pyogenes* quorum sensing systems that control various virulence attributes are categorized into the LuxS/AI-2, Sil, lantibiotics, and Rgg systems^9^,^10^. Canonically, the bacteria detect the quorum sensing molecule that they synthesize and thus generate a coordinated response^11^. However, in a polymicrobial community, several species of bacteria have been shown to detect the signaling molecules that they did not synthesize, a process known as eavesdropping^12^,^13^,^14^. Through the process of interspecies signaling, bacteria can sense the surrounding populations in a polymicrobial environment. Quorum sensing is an essential part of the interspecies competition, and the process of interspecies signaling/eavesdropping keeps competitors in check in a polymicrobial setting^15^. Therefore, interspecies strategies that interfere with quorum sensing signals can be explored to develop new-generation antimicrobials. In addition, data on interspecies quorum sensing signaling interference will provide insights into bridging the gap in knowledge regarding asymptomatic colonization.

The secretion of AHLs and use of quorum sensing molecules in the regulation of various virulence phenotypes have been reported for Gram-negative bacteria^16^. However, a documented role for AHLs in the pathogenesis of Gram-positive bacteria is lacking. Therefore, the present study aimed to investigate the role of AHLs in the regulation of *S. pyogenes* M6 S165 virulence and growth.

## Results

### AHLs inhibit the hemolytic activity of *S. pyogenes* M6 S165

**Figure 1:**
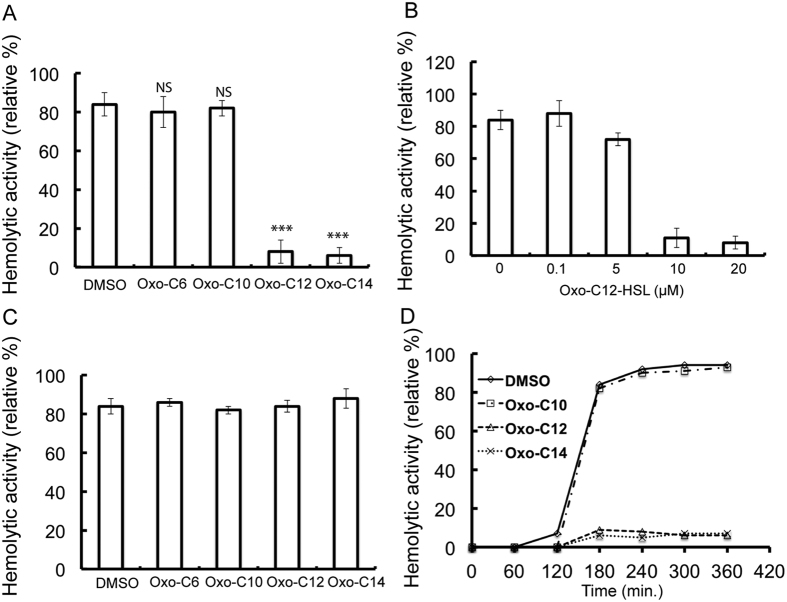
Effect of oxo-AHL on the hemolytic activity of *S. pyogenes.* (**A**) Hemolytic activity of *S. pyogenes* M6 S165 grown in the presence of different oxo-AHLs (20 μM). (**B**) Hemolytic activity of *S. pyogenes* M6 S165 co-incubated with different concentrations of oxo-C12-HSL. (**C**) *S. pyogenes* M6 S165 was grown in the presence of varying concentrations of oxo-C12-HSL, and the supernatant from the growth was analyzed for hemolytic activity. (**D**) Hemolytic activity of *S. pyogenes* M6 S165 in the presence of inhibitory oxo-AHLs during different growth stages. The hemolytic activity is measured in relative to caused by water.

The present study investigated the effect of AHLs on the hemolytic activity of *S. pyogenes* M6 S165. *S. pyogenes* M6 S165 was cultured in the presence of different AHLs, which varied in the number of carbon atoms in their fatty acid side chain, and then assayed for hemolytic activity. The AHLs with fatty acid side chains containing ≥12 carbon atoms significantly (P < 0.001) inhibited the hemolytic activity of *S. pyogenes* M6 S165 (Fig. 1A). The inhibitory effect of the AHLs fell within the micromolar concentration range (Fig. 1B). The SLS-mediated hemolytic activity was confirmed using trypan blue as SLS gets inhibited in the presence of trypan blue^17^. The hemolytic activity noted in the growth supernatant was completely abolished by the addition of trypan blue (Supplementary Figure S1), indicating that streptolysin S (SLS) was the primary erythrolysin present in the supernatant. To determine if the inhibitory AHLs inactivated the SLS secreted in the growth media, the supernatant from the growth of *S. pyogenes* M6 S165 was mixed with different AHLs (20 μM) and assayed for hemolytic activity. A noticeable decrease in hemolytic activity was not observed, suggesting that AHLs did not inactivate the secreted SLS (Fig. 1C). Furthermore, the reduction in the *S. pyogenes* M6 S165 hemolytic activity due to AHLs could not be attributed to a defect in the growth rate (Supplementary Figure S1). The decrease in hemolytic activity by the inhibitory AHLs was not transient, but rather it was observed throughout the growth phase (Fig. 1D). These results indicate that observed inhibitory effects of oxo-C12-HSL and oxo-C14-HSL on hemolytic activity was not through inactivation of secreted SLS.

### AHLs interfere with the expression of the *sag* operon

**Figure 2:**
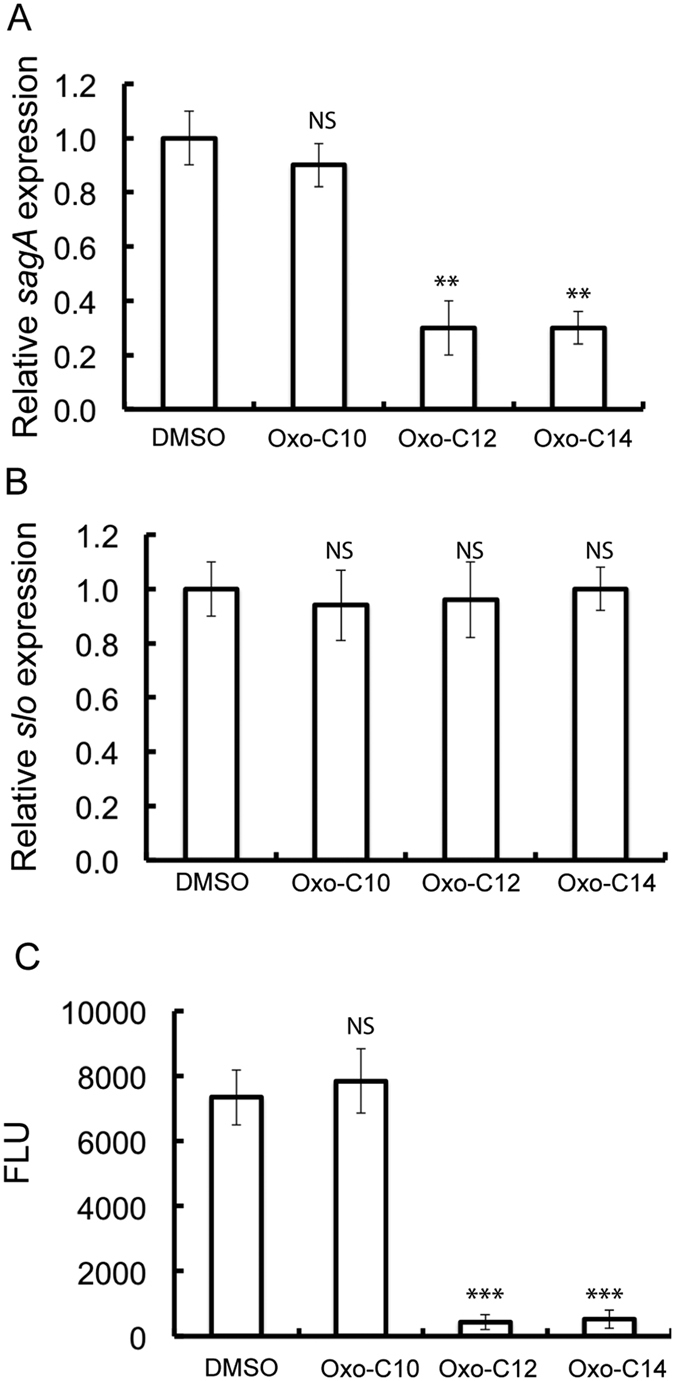
Oxo-AHLs inhibit transcription of *saga.* (**A**) Expression of *sagA* during co-incubation with inhibitory oxo-AHLs. (**B**) Expression of *slo* during co-incubation with inhibitory oxo-AHLs. (**C**) Luciferase assay to determine the effect of inhibitory oxo-AHLs on the promoter activity of *sagA*.

For group A *Streptococcus*, the contiguous nine-gene *sag* operon (*sagABCDEFGHI*) encodes the functional SLS^18^,^19^. The effect of AHLs on the expression of the *sag* operon was monitored by qPCR. A significant (P < 0.05) decrease in the expression of *sagA* was observed when *S. pyogenes* M6 S165 was cultured in the presence of oxo-C12-HSL (10 μM) and oxo-C14-HSL (10 μM) (Fig. 2A). However, the *sagA* expression was not altered when *S. pyogenes* M6 S165 was cultured in the presence of oxo-C10-HSL. Thus, a decrease in the *sagA* transcripts was detected only in *S. pyogenes* M6 S165 cultured with the inhibitory AHLs. The transcript levels of the *slo* gene encoding streptolysin O remained unaffected (Fig. 2B), thereby indicating that the inhibition of the hemolytic activity due to the oxo-AHLs is the result of a decrease in SLS. To further determine if oxo-C12-HSL and oxo-C14-HSL inhibited the expression of SLS at the transcriptional level, a promoter assay utilizing a fusion of the promoter region of the *sag* operon to a promoterless luciferase reporter gene was conducted^20^. A significant (P < 0.001) decrease in luciferase activity was observed for the luciferase gene-containing *S. pyogenes* M6 S165 cultured in the presence of oxo-C12-HSL (10 μM) and oxo-C12-HSL (10 μM) (Fig. 2C). These results suggested that oxo-C12-HSL and oxo-C14-HSL inhibit the hemolytic activity of *S. pyogenes* M6 S165 by regulating the expression of the *sag* operon.

### *P. aeruginosa* growth supernatant inhibits the hemolytic activity of *S. pyogenes* M6 S165

**Figure 3:**
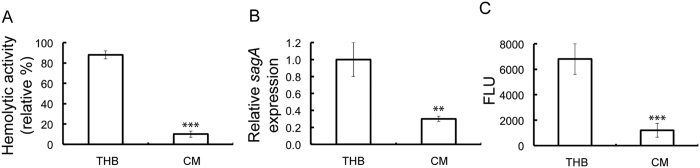
*P. aeruginosa* growth supernatant inhibits the hemolytic activity of *S. pyogenes.* (**A**) Hemolytic activity of *S. pyogenes* M6 S165 grown in THB and CM obtained from growth of *P. aeruginosa*. (**B**) Expression of *sagA* in S*. pyogenes* M6 S165 grown in THB and CM obtained from growth of *P. aeruginosa*. (**C**) Luciferase assay to determine the effect of CM on the promoter activity of *sagA*.

*P. aeruginosa* has been shown to secrete AHLs with varying fatty acid side chains. Conditioned media (CM) was prepared from the growth supernatant of *P. aeruginosa*^21^,^22^,^23^. The growth of *S. pyogenes* M6 S165 was not affected in the CM (Supplementary Figure S3). A significant (P < 0.001) decrease in the hemolytic activity of *S. pyogenes* M6 S165 grown in CM compared to *S. pyogenes* M6 S165 grown in THB was observed (Fig. 3A). The expression of *sagA* was significantly (P < 0.05) reduced in *S. pyogenes* M6 S165 cultured in CM compared to *S. pyogenes* M6 S165 cultured in THB (Fig. 3B). In addition, a significant (P < 0.001) reduction in the luciferase activity of *S. pyogenes* M6 S165 harboring the *sagA* promoter fused to the luciferase reporter gene was observed when *S. pyogenes* M6 S165 was cultured in CM (Fig. 3C). Therefore, the CM obtained from the growth of *P. aeruginosa* inhibits the SLS-mediated hemolytic activity of *S. pyogenes* M6 S165.

### Inhibition of the hemolytic activity due to AHLs is strain-specific

**Figure 4:**
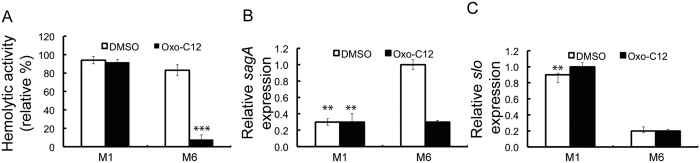
Strain specificity in the inhibition of hemolytic activity due to oxo-AHLs. (**A**) Hemolytic activity of two different strains of *S. pyogenes* in the presence of oxo-AHLS. (**B**) Expression of *sagA* in *S. pyogenes* M1 and M6 during co-incubation with oxo-C12-HSL. (**C**) Expression of *slo* in *S. pyogenes* M1 and M6 during co-incubation with oxo-C12-HSL.

The effect of AHLs on the hemolytic activity of *S. pyogenes* M1 strain was examined and no decrease was recorded in the presence of oxo-C12-HSL (10 μM) (Fig. 4A). In addition, the *sagA* transcript levels of *S. pyogenes* M1 grown in the presence of oxo-C12-HSL (10 μM) remained unaffected (Fig. 4B). The change in the expression of *sagA* due to oxo-C12-HSL (10 μM) was observed only for *S. pyogenes* M6 S165. However, the transcript levels of *sagA* were significantly (P < 0.05) lower in *S. pyogenes* M1 compared to those of *S. pyogenes* M6 S165 (Fig. 4B). In addition, a significantly (P < 0.05) higher amount of *slo* transcripts were detected in *S. pyogenes* M1 compared to the amount detected in *S. pyogenes* M6 S165 (Fig. 4C). The transcript level of *slo* remained unaffected for both strains grown in the presence of oxo-C12-HSL (10 μM). Altogether, these results demonstrated that the *slo*-mediated hemolytic activity predominates in *S. pyogenes* M1 and is unaffected by AHL. Therefore, the AHL-mediated inhibition of the hemolytic activity can be attributed to the reduced expression of SLS.

### AHLs influence the expression of *luxR* and the intracellular iron concentration

**Figure 5:**
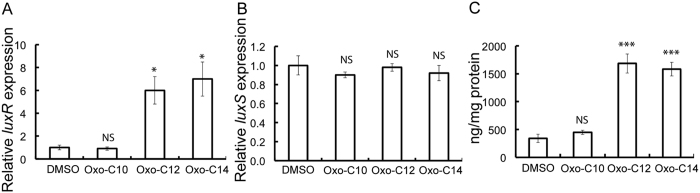
Effect of oxo-AHLs on the transcriptional activity of *luxR* and *luxS* and the total intracellular iron content. (**A**) Expression of *luxR* during co-incubation with different oxo-AHLs. (**B**) Expression of *luxS* during co-incubation with different oxo-AHLs. (**C**) Total intracellular iron content during co-incubation with different oxo-AHLs.

The role of AHLs in the activation of the transcriptional regulator LuxR and influx of iron was examined. It has been demonstrated that expression and activity of LuxR family of transcriptional regulators are affected by AHLs^24^. Therefore, we investigated if the AHLs have an influence on the expression of *luxR*. The genome sequence of strain *S. pyogenes* MGAS 10394 from the NCBI database was used for reference. The gene M6_Spy1777 has been annotated as LuxR in MGAS 10394 and some other *S. pyogenes* strains, the protein has DNA binding domain. It has been shown that AHLs generate the dissociation product, tetramic acid, with an ability to bind iron^25^. Therefore we speculate that AHLs could result in increased levels of intracellular iron. The expression of *luxR* and *luxS* in *S. pyogenes* M6 S165 was assessed by qPCR. A significant (P < 0.05) increase in the expression of *luxR* was observed in *S. pyogenes* M6 S165 grown in the presence of oxo-C12-HSL (10 μM) and oxo-C14-HSL (10 μM) (Fig. 5A), whereas the presence of oxo-C10-HSL (10 μM) had no effect on *luxR* expression. The expression levels of *luxS* remained unaltered in *S. pyogenes* M6 S165 grown in the presence of any of the AHLs (Fig. 5B). A marked (P < 0.001) increase in the intracellular iron concentration in *S. pyogenes* M6 S165 grown in the presence of oxo-C12-HSL (10 μM) and oxo-C14-HSL (10 μM) was observed (Fig. 5C). These results demonstrate an increase in the transcriptional activity of *luxR* in addition to a heavy influx of intracellular iron.

### Role of LuxR in the AHL-mediated inhibition of SLS activity

**Figure 6:**
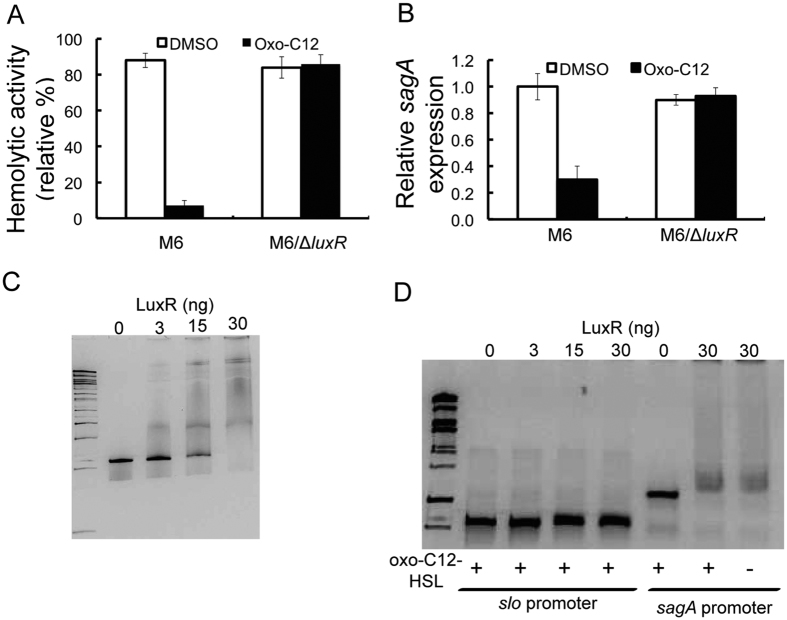
Role of *luxR* in the oxo-AHL-mediated inhibition of SLS activity. (**A**) Hemolytic activity of *S. pyogenes* M6 S165 and its *luxR* mutant grown in the presence of oxo-C12-HSL. (**B**) Expression of *sagA* in *S. pyogenes* M6 S165 and its *luxR* mutant grown in the presence of oxo-C12-HSL. (**C**) EMSA to demonstrate the binding of luxR to the promoter region of *sagA*. (**D**) EMSA to demonstrate the bindiding of luxR is specific to DNA from the promoter region of *sagA* and not affected by AHL. No gel shift for DNA from *slo* promoter is observed when incubated with oxo-C12-HSL (10 μM). Also, *sagA* promoter a gel shift was observed even in the absence of oxo-C12-HSL.

To verify the role of LuxR in the regulation of the *sag* operon, an insertional mutant construct for the *luxR* gene was established in *S. pyogenes* M6 S165. The hemolytic activity of the *luxR* mutant strain grown in the presence of oxo-C12-HSL remained unaffected (Fig. 6A). Moreover, the transcript levels of *sagA* in the *luxR* mutant strain grown in the presence of oxo-C12-HSL were unaltered (Fig. 6B). The role of *luxR* in the regulation of the *sag* operon was also examined by EMSA. The 334 bp probe contained the sequence of the upstream segment of the initiation sequence of *sagA.* Purified LuxR was found to interact with the promoter region of *sagA* (Fig. 6C). No interaction of LuxR with the DNA from slo promoter region was detected from the gel shift assay (Fig. 6D). It was also found that oxo-C12-HSL was not required for binding of LuxR to DNA from the promoter region of sagA (Fig. 6D). Furthermore, when luxR was expressed under a constitutive promoter of *gyrA* inhibition in the hemolytic activity was observed (Supplementary Figure 4). However, there was a slight reduction in growth of the *S. pyogenes* M6 165 strain harboring the *luxR* construct under *gyrA* promoter (Supplementary Figure S4). Thus, LuxR negatively regulates the expression of the *sag* operon.

### Higher concentrations of AHLs inhibit the growth of *S. pyogenes* M6 S165

**Figure 7:**
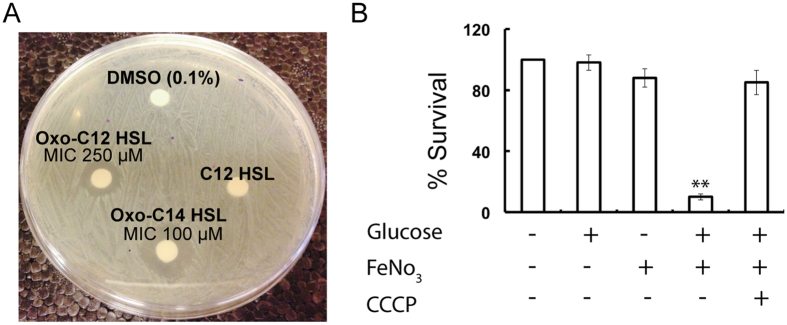
Bactericidal effect of oxo-AHLs. (**A**) Disk diffusion assay to demonstrate the inhibition of *S. pyogenes* M6 S165 growth by the oxo-AHLs. (**B**) Effect of glucose and iron on the *S. pyogenes* M6 S165 growth inhibition by oxo-C12-HSL.

The minimum inhibitory concentrations (MICs) of AHLs against *S. pyogenes* M6 S165 were determined by the serial dilution assay. The MICs of oxo-C12-HSL and oxo-C14-HSL were 250 and 100 μM, respectively. The disk diffusion assay was used to analyze the effect of AHLs on *S. pyogenes* M6 S165 growth. Oxo-C12-HSL (250 μM) and oxo-C14-HSL (100 μM) inhibited the growth of *S. pyogenes* M6 S165 (Fig. 7A), while C12-HSL (250 μM) did not exhibit growth inhibition. This suggests that the antimicrobial effect was not due to the fatty acid side chain. Therefore, to determine if Oxo-C12-HSL exert bactericidal effect on *S. pyogenes* M6 S165 a survival assay was performed. A survival assay, which used PBS, was then conducted to assess the conditions required for the bactericidal activity of oxo-C12-HSL (Fig. 7B). Oxo-C12-HSL (1 mM) in PBS alone was not found to be lethal to *S. pyogenes* M6 S165. Moreover, in the presence of either glucose or FeNO_3_, the survival of *S. pyogenes* was unaffected by oxo-C12-HSL (1 mM). However, in the case of co-incubation with both glucose and FeNO_3_, the survival of *S. pyogenes* M6 S165 decreased by 95%. The addition of carobonyl cyanide m-chlorophenyl hydrazine (CCCP), a chemical inhibitor of oxidative phosphorylation, abrogated the bactericidal activity of oxo-C12-HSL even when PBS was supplemented with both glucose and FeNO_3_, thereby indicating that the bactericidal activity of oxo-C12-HSL was dependent on the membrane potential.

Similar results were also obtained by the M1 strain of *S. pyogenes* (Supplementary figure S2). Thus, high concentrations of AHLs inhibit the growth of both *S. pyogenes* M1 and M6 strains reliant on the availability of glucose and iron.

### Ferrichrome transporter FtsABCD is involved in the uptake of AHLs by *S. pyogenes* M6 S165

**Figure 8:**
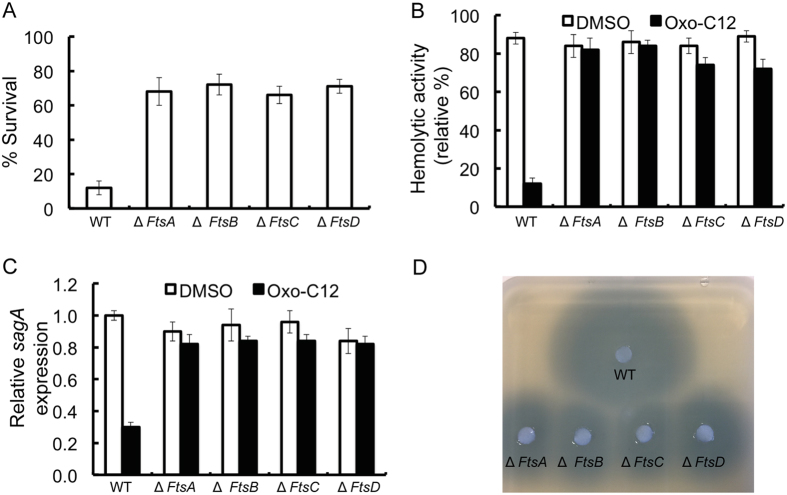
Role of the ferrichrome transporter in the oxo-AHL-mediated killing of *S. pyogenes.* (**A**) Survival assay of *S. pyogenes* M6 S165 and its ferrichrome transporter mutants in the presence of oxo-C12-HSL. (**B**) Hemolytic activity of *S. pyogenes* M6 S165 and its ferrichrome transporter mutants grown in the presence of oxo-C12-HSL. (**C**) Expression of *sagA* in *S. pyogenes* M6 S165 and its ferrichrome transporter mutants grown in the presence of oxo-C12-HSL. (**D**) Bioassay to determine the intracellular accumulation of oxo-C12-HSL in *S. pyogenes* M6 S165 and its ferrichrome transporter mutants grown in the presence of oxo-C12-HSL.

To investigate the transporter involved in the uptake of AHLs, we constructed an insertional mutants for the ferrichrome transporter FtsABCD in *S. pyogenes* M6 S165. The bactericidal effect of oxo-C12-HSL (1 mM) was nullified in all the *S. pyogenes* M6 S165 FtsABCD mutants (Fig. 8A). The inhibitory effect of oxo-C12-HSL on hemolytic activity was also lost in all of the *S. pyogenes* M6 S165 FtsABCD mutants (Fig. 8B). Additionally, transcript levels of *sagA* in the *S. pyogenes* M6 S165 FtsABCD mutants grown in the presence of oxo-C12-HSL were unchanged (Fig. 8C). An *Agrobacterium tumefaciens*-based bioassay was conducted to estimate the intracellular concentration of oxo-C12-HSL in the wild type *S. pyogenes* and FtsABCD mutants cultured in the presence 20 μM oxo-C12-HSL (Fig. 8D). The bioassay revealed the presence of lower intracellular amounts of oxo-C12-HSL in the FtsABCD mutants compared to concentrations present in the wild type. This data suggests a potential role for FtsABCD in the uptake of AHLs.

## Discussion

The current study aimed to investigate the role of AHLs, a group of quorum sensing molecules released by several members of the host microbiota, in the virulence of *S. pyogenes*. We demonstrated that the AHLs with fatty acid side chains of ≥12 carbon atoms inhibit *S. pyogenes* M6 S165 SLS-mediated hemolytic activity by negatively regulating the expression of the *sag* operon. Furthermore, the study revealed that the transport of AHLs across the streptococcal cell membrane is an energy driven process facilitated by the ferrichrome transporter FtsABCD.

*S. pyogenes* encodes SLS, which is a non-immunogenic post-translationally modified secreted virulence peptide with the ability to lyse mammalian erythrocytes^19^. In addition, SLS impairs the membranes of lymphocytes, neutrophils, platelets, lysosomes, and mitochondria^26^,^27^,^28^,^29^, clearly playing a vital role in streptococcal virulence. We, therefore, investigated the role of AHLs in the expression of SLS. In the present study, we found that SLS was the primary hemolysin secreted into the growth media, and its expression was detected towards the latter part of the exponential phase, indicating a cell-density-dependent phenomenon. A similar cell density-dependent expression of SLS was previously reported and speculated to act as a quorum sensing molecule in *S. pyogenes*^30^,^31^. The SLS-mediated hemolytic activity was completely abolished only when *S. pyogenes* M6 S165 was cultured in media containing oxo-C12-HSL or oxo-C14-HSL. The presence of oxo-C6-HSL or oxo-C10-HSL in the growth media had no inhibitory effect on hemolytic activity. The data from the qPCR analysis and transcriptional fusion assays revealed that the inhibitory AHLs decreased the hemolytic activity by negatively regulating the promoter activity of the *sag* operon, thereby downregulating the expression of SLS. The inhibitory effect of the AHLs was found to be specific towards SLS, as revealed by the data obtained from the *S. pyogenes* M1 strain, which predominantly produced SLO in the THB growth media used in the hemolytic assay experiments. The *sag* operon is highly conserved among group A *Streptococcus*^19^. Therefore, ambiguity in the strain-specific secretion of SLS into the THB growth media remains unresolved and requires further investigation. Together, this study provides experimental evidence that the AHLs with fatty acid side chains of ≥12 carbon atoms specifically inhibit (within the micromolar range) the SLS-mediated hemolytic activity of *S. pyogenes* M6 S165 without altering the growth rate. A similar effect of AHLs with fatty acid side chains of ≥12 carbon atoms has been reported in *Staphylococcus aureus* wherein AHLs interact with the cytoplasmic membrane and downregulate the exotoxin production and *agr* mediated quorum sensing^32^.

The observations suggest a scenario wherein *S. pyogenes,* when colonizing in the vicinity of microbes capable of secreting AHLs with fatty acid side chains of ≥12 carbon atoms, will be attenuated in its ability to produce the virulence marker SLS. *P. aeruginosa* is reported to secrete a variety of AHLs including oxo-C12-HSL and oxo-C14-HSL^21^,^22^. In addition, *P. aeruginosa* is a frequent colonizer of the respiratory tract, identified as the causative agent of chronic lung infections and the most prevalent pathogen associated with cystic fibrosis^2^,^33^. Therefore, it was speculated that the CM obtained from the growth of *P. aeruginosa* should negatively affect the hemolytic activity of *S. pyogenes*. To confirm this hypothesis, the supernatant from the culture of *S. pyogenes* M6 S165 in CM was analyzed for hemolytic activity. The CM from *P. aeruginosa* was able to inhibit *S. pyogenes* M6 S165 SLS-mediated hemolytic activity by downregulating the expression of *sagA*.

To gain mechanistic insights into the inhibition of SLS-mediated hemolytic activity by AHLs, the roles of *luxR, luxS*, and the intracellular iron concentration were examined. One of the mechanisms in bacterial quorum sensing involves the synthesis of the autoinducers through the evolutionarily conserved LuxI family of autoinducer synthases and its homologs, while the responses towards particular autoinducers are generated through the evolutionary conserved LuxR family of transcriptional regulators^24^,^34^. In *S. pyogenes, luxS* is involved in the synthesis of the quorum sensing molecule autoinducer 2^35^. In the present study, an increase in the transcript levels of *luxR* was observed for *S. pyogenes* M6 S165 grown in the presence of oxo-C12-HSL and oxo-C14-HSL. However, no effect on the expression of *luxS* was observed. The SLS-mediated hemolytic activity and expression of *sagA* remained unaffected in the *S. pyogenes* M6 S165 *luxR* mutant. EMSAs revealed that LuxR could potentially bind to the region upstream of *sagA*, thereby affecting its promoter activity. Hence, we hypothesize that the inhibitory AHLs induce the expression of the transcriptional regulator LuxR which in turn negatively regulates the *sag* operon. The production of SLS in *S. pyogenes* is reportedly influenced by the concentration of iron in the growth medium^36^; however, the underlying mechanism has yet to be elucidated. Nevertheless, a role for SLS in the acquisition of iron has been proposed^37^,^38^. We observed a 3-fold increase in the *S. pyogenes* M6 S165 intracellular iron concentration grown in the presence of inhibitory AHLs. Hence, the AHL-mediated inhibition of hemolytic activity might be the result of an excess accumulation of intracellular iron.

The growth of *S. pyogenes* M6 S165 was not affected by either oxo-C12-HSL or oxo-C14-HSL when used in micromolar quantities. However, at higher concentrations, bactericidal effects were observed. A similar bactericidal activity of AHLs affecting Gram-positive bacteria has been reported by Kaufmann *et al*.^25^. Davis *et al*. have demonstrated that oxo-C12-HSL and oxo-C14-HSL are capable to interact with the cellular membrane in the micromolar range and cause changes in the membrane dipole potential^39^. The survival assay demonstrated that the antibacterial effect of oxo-C12-HSL on *S. pyogenes* M6 S165 was an energy-driven process with an absolute requirement for glucose and iron. The AHLs generate the dissociation product, tetramic acid, with an ability to bind iron^25^. In *S. pyogenes*, the ABC transporter FtsABCD has been implicated as a transporter involved in the uptake of Fe^3+^ ferrichrome^40^. Therefore, we sought to determine if FtsABCD participates in the uptake of oxo-C12-HSL. Although, the bioassay was qualitative it provides clue that the ferrichrome transporter FtsABCD was involved in the transport of the oxo-C12-HSL across the membrane. Moreover, FtsABCD mutants were insensitive towards AHL-mediated inhibition of hemolytic activity and killing. The results indicate a potential interaction of oxo-C12-HSL with iron to form a complex, with subsequent uptake facilitated by the ferrichrome transporter FtsABCD.

In summary, this study provides evidence for the role of AHLs in the virulence of the Gram-positive bacteria *S. pyogenes* M6 S165. We demonstrated that the inhibitory effect of the AHLs on hemolytic activity was due to LuxR-mediated downregulation of the *sag* operon. In addition, the ferrichrome transporter FtsABCD facilitated the transport of AHLs across the *S. pyogenes* membrane. However the study is limited with its results based on single strain, it will be intriguing to further investigate differences in SLS/SLO expression profiles in different *S. pyogenes* strains and the resultant hemolytic activity as affected by different quorum sensing molecules such as AHLs. These intriguing results highlight the importance of additional studies needed to elucidate the role of inter-bacterial communications in the expression of virulence markers.

## Methods

### Bacterial strains and growth conditions

The clinical *S. pyogenes* isolates S165 (*emm6*) and S291 (*emm1*) are blood isolates from patients with severe invasive streptococcal disease^41^. The strain *Agrobacterium tumefaciens* NTL4 (pZLR4) used in AHL bioassays was a kind gift from Prof. Stephen Farrand at the University of Illinois, USA. *S. pyogenes* was grown on GC agar (Acumedia; Lansing, MI, USA). Liquid cultures of *S. pyogenes* were grown in Todd Hewitt Broth (THB, Acumedia) at 37 °C and 5% CO_2_. *A. tumefaciens* NTL4 (pZLR4) was grown on Luria Agar (Acumedia) with 30 μg/mL of gentamicin at 28 °C. The AHLs (Sigma-Aldrich) oxo-C6-HSL, oxo-C10-HSL, oxo-C12-HSL, and oxo-C14-HSL were added at varying concentration to the THB. *Pseudomonas aeruginosa* PA01 was a kind gift from Dr. Klaus Udekwu at the Stockholm University, Sweden. The genome sequence of strain *S. pyogenes* MGAS 10394 from the NCBI database was used for reference in construction of the primers employed in this study.

### Hemolytic activity

The assay for hemolytic activity was performed as described previously^20^. Horse blood (Håtunalab AB, defibrinated) was used to obtain red blood cells (RBCs). Briefly, *S. pyogenes* was cultured in THB with or without 5 μM AHLs from A_600_ ≈ 0.1 to A_600_ ≈ 1.0. The cells were harvested by centrifugation; the supernatant obtained was passed through a 0.2 μm filter and used for the hemolytic assay. The filtrate was diluted 1:10 in THB, mixed with RBCs at a 1:1 ratio, and incubated at 37 °C in 5% CO_2_ for 1 h. The hemolysis was estimated at A_404_, and the activity is reported as the percentage relative to water alone. For the hemolytic assays describing the non-transient inhibitory activity of AHLs, the supernatant was obtained every hour until the growth reached the stationary phase.

### Quantitative PCR assays

*S. pyogenes* was cultured in THB with or without AHLs from A_600_ ≈ 0.1 to A_600_ ≈ 1.0. The cells were harvested by centrifugation, treated with mutanolysin for 1 h at 37 °C, and subjected to total RNA isolation with the RNeasy Mini Kit (Qiagen). The cDNA was synthesized from 200 ng RNA using SuperScript VILO^TM^ master mix (ThermoFisher Scientific). The primers used for the quantification of *sagA, luxS, luxR*, and *gyrA* transcripts are listed in Supplementary Table 1. The PCR reaction conditions were as follows: an initial denaturation step at 95 °C for 10 min and 45 cycles at 95 °C for 15 s and 60 °C for 1 min. The fold change was calculated relative to the housekeeping gene *gyrA*.

### Luciferase assay

The construction of the transcriptional fusion of the *sagA* promoter to the promoterless firefly luciferase gene used in the present study was previously reported^20^. The *S. pyogenes* M6 strain harboring this construct was grown in THB from A_600_ ≈ 0.1 to A_600_ ≈ 1.0 with or without AHLs, and the luciferase activity was measured using the Luciferase Assay Kit (Promega) according to the manufacturer’s instructions.

### Conditioned media (CM)

The CM from the growth of *P. aeruginosa* was prepared as described previously^42^. *P. aeruginosa* was grown in THB at an initial density of A_600_ ≈ 0.1 until the culture reached A_600_ ≈ 1.0. The cells were harvested by centrifugation and the supernatant was filtered through a 0.2 μm filter. The filtrate was adjusted to pH 7.8 and used as CM.

### *S. pyogenes* M6 S165 gene manipulation

The *S. pyogenes* M6 S165 mutants for *slo, luxR, FtsA, FtsB, FtsC*, and *FtsD* were constructed by directed insertional inactivation using the pSPC18 plasmid as described by Lyon *et al*.^43^. Briefly, the internal region of interest in the gene was amplified and inserted into the *BamHI* site of pSPC18. The construct was electroporated into *S. pyogenes* M6 S165 to obtain the desired gene disruption via homologous recombination, which was confirmed by PCR. The strains were then maintained with 30 μg/mL spectinomycin. For construction of *luxR* under *gyrA* promoter, a fusion PCR was performed with the luxR coding sequence and 350 bp fragment upstream of *gyrA*. The resultant product was inserted into *BamHI* and *NdeI* site of the *Streptococcus* shuttle vector PJRS525 and electroporated *to S. pyogenes* M6 S165^44^. The primers used for gene amplification are listed in Supplementary Table 2.

### Measurement of intracellular iron

*S. pyogenes* M6 was cultured in THB with or without AHLs. The cells were harvested at A_600_ ≈ 1.0 and subjected to intracellular iron measurements using the Iron Assay Kit (Sigma-Aldrich) according to manufacturer’s instructions. The values represent the total iron content in ng/mg protein. The total protein content was estimated by Bradford assay.

### Electrophoretic mobility shift assay (EMSA)

LuxR was His-tagged at the N-terminal using the pTrcHis TOPO^®^ TA Expression Kit (Invitrogen, USA). For cloning, a 3075 bp region consisting of the *luxR* ORF was amplified using the primers luxR-his F 5′-ATGACGAAGGGTATTCGATTTC-3′ and luxR-his R 5′-TTAGTTGTTAGAGGAGAATTGC-3′. The tagged LuxR was purified using standard Ni-NTA agarose (Qiagen) purification protocols. The purity of the protein was assessed by standard SDS-PAGE, and the purified protein was stored in 15% glycerol at −80 °C.

For the EMSAs, a 334 bp region upstream of the *sagA* start codon was amplified with primers sagA-Shift-F 5′-GGATGAAGTAAAGATATTAGCTAGGG-3′ and sagA-Shift-R 5′-GGTTTACCTCCTTATCTAATAAGTAAC-3′ and a 273 bp region upstream of *slo* was amplified with primers slo-Shift 5′ ACCCAATTGAAAGCTAACATCG-3′ and slo-Shift-R 5′-TGTTCTTTCGACCATATCAAGCA-3′. The product was purified using DNA Clean & Concentrator™-25 (Zymo Research, USA). The DNA binding assay was conducted in binding buffer (20 mM Tris-HCl, pH 7.4; 50 mM KCl; 1 mM EDTA; 1 mM DTT; 5% glycerol; and 100 μg/mL BSA) as described previously^45^. The binding buffer was supplemented with 10 μM oxo-C12-HSL. The reactions consisted of 50 ng purified DNA and increasing amounts of N-terminal His-tagged LuxR (3–30 ng).

### Survival assay

*S. pyogenes* was grown in THB and harvested at the mid-exponential phase. The cells were washed three times with PBS and resuspended in PBS at a cell density of 10^7^ cfu/mL; 100 μL of the cell suspension was incubated with 1 mM oxo-C12-HSL in PBS at 37 °C for 3 h. Glucose was added at a final concentration of 0.4%. FeNO_3_ was used at a final concentration of 25 μM. Following incubation, the suspension was serially diluted and plated on GC agar to determine viability. The minimum inhibitory concentrations for the AHLs were determined by standard disk diffusion assays.

### AHL bioassay

The bioassay to measure the uptake of AHLs by *S. pyogenes* was performed using the *A. tumefaciens* NTL4 (pZLR4) strain. Although the *A. tumefaciens* NTL4 (pZLR4) strain does not produce AHLs, it responds to a variety of AHLs^46^. *S. pyogenes* was grown in THB with or without 10 μM oxo-C12-HSL. The cells were harvested at a density of A_600_ ≈ 1.0, treated with mutanolysin at 37 °C for 1 h, and lysed by sonication. Next, 10 μL of the lysate after centrifugation was applied to Whatman filter disks and allowed to dry. 500 μl of the overnight growth of *A. tumefaciens* NTL4 (pZLR4) was added to LBA cooled to 50 °C supplemented with 25 μg/mL gentamicin and poured on standard petri dishes. The plate was overlaid with 0.7% bacteriological agar containing Bluo-Gal (Sigma-Aldrich). The disk with the lysate was placed on the overlaid agar. The plate was incubated at 37 °C for 16 h, and the precipitation zone due to β-galactosidase activity was measured.

### Statistical analysis

All experiments were performed in triplicate and repeated three times. Analysis of variance (ANOVA) and the Student’s t-test were employed to analyze the difference between the groups for statistical significance. P < 0.05 was considered statistically significant. The data is represented as the mean ± standard deviation. The stars in the bar graph denote statistical significance.

## Additional Information

**How to cite this article:** Saroj, S. D. *et al*. Inhibitory role of acyl homoserine lactones in hemolytic activity and viability of *Streptococcus pyogenes* M6 S165. *Sci. Rep.* **7**, 44902; doi: 10.1038/srep44902 (2017).

**Publisher's note:** Springer Nature remains neutral with regard to jurisdictional claims in published maps and institutional affiliations.

## Supplementary Material

Supplementary Data
