# Supplementary material for: Inhibitory role of acyl homoserine lactones in hemolytic activity and viability of Streptococcus pyogenes M6 S165
Source: Sci Rep. 2017 Mar 17;7:44902. doi: 10.1038/srep44902 (PMC5355980; doi:10.1038/srep44902)

Inhibitory role of acyl homoserine lactones in hemolytic activity and viability of *Streptococcus pyogenes* M6 S165

Sunil D. Saroj, Linda Holmer, Júlia M. Berengueras, and Ann-Beth Jonsson*

Department of Molecular Biosciences, The Wenner-Gren Institute, Stockholm University, 10691 Stockholm, Sweden.

***Correspondence:**

Ann-Beth Jonsson, E-mail: ann-beth.jonsson@su.se, Phone: +46 8 164154

Running title: AHLs interfere with GAS virulence

Keywords: *Streptococcus pyogenes*, Acyl homoserine lactone, streptolysin, microbiota, quorum sensing

Supplementary Table 1. List of primers used for qPCR

Gene	Sequence	
sagA	F	5′-GCTACTAGTGTAGCTGAAACAACTCAA-3′
	R	5′-AGCAACAAGTAGTACAGCAGCAA-3
luxS	F	5'-AAGCTAATCCGCCAACGCAT-3'
	R	5'-TTTGGCGGCAAAGAGGCTAT-3'
luxR	F	5'-GCAGGCGTTAAAGGCACAAA-3'
	R	5'-CTTTGATGAGTCAGGGGCGT-3'
gyrA	F	5′-CGACTT GTCTGAACGCCAAAGT-3′
	R	5′-ATCACGTTCCAAACCAGTCAAAC-3′
slo	F	5'-TTGTTGAGGATAATGTAAGAATGTTTAG-3'
	R	5'-TCCTGGCTTGCAACTGATTG-3'
covS	F	5'-CATCTCCTGGCTTGCATGGT-3'
	R	5'-GGAAAACCCACGATACTGATCTTC-3'

Supplementary Table 2. List of primers used for construction of *S. pyogenes* M6 S165 mutant strains

Gene	Sequence	
slo	F	5'-ATGAAAGGATCCCCGCCACTCTTTGTGAG-3'
	R	5'-AAGTTACTGCAGCGATCACTTTTCGCCAC-3'
luxR	F	5'-ATGAAAGGATCCCCGTTTGGCAATTTGTCCGT-3'
	R	5'-AAGTTACTGCAGCTTTGATGAGTCAGGGGCGT-3'
FtsA	F	5'-ATGAAAGGATCCGGCCGCTAAGCCAATTGATG-3'
	R	5'-AAGTTACTGCAGCTAGGAGGCGGTTCTCTTGC-3'
FtsB	F	5'-ATGAAAGGATCCTGCCAGCCGATAACAGCATT-3'
	R	5'-AAGTTACTGCAGGCAAAACGGCCATAAAGCCA-3'
FtsC	F	5'-ATGAAAGGATCCCCACGTCCCCAGTCTTTACC-3'
	R	5'-AAGTTACTGCAG TGCCTCAAATTGCTGGTGTG-3'
FtsD	F	5'-ATGAAAGGATCCCAAACCCTTTGACGTTGCCC-3'
	R	5'-AAGTTACTGCAGGCTGAAGACCTCACAATTGCC-3'
luxR-P	F	5'- ATTCCTTAAAATGACGAAGGGTATT-3'
	R	5'- CCTCATATGAGTTAGTTGTTAGAGGAGAA-3'
gyrA-P	F	5'- CCTGGATCCGCAATATTTTGTGTTACC-3'
	R	5'- CCTTCGTCATTTTAAGGAATGTCCTTTTC-3'

Supplementary Figure S1. Concentration of oxo-C12-HSL that inhibits hemolytic activity, and an assay to determine the type of hemolysin produced

(A) Growth curve of *S. pyogenes* M6 S165 in the presence of different oxo-AHLs (20 μM) (B) *S. pyogenes* M6 S165 growth supernatant was incubated with trypan blue and subjected to a hemolytic assay. The hemolytic activity was suppressed in the presence of trypan blue, indicating that SLS is the major hemolysin released into the growth media.

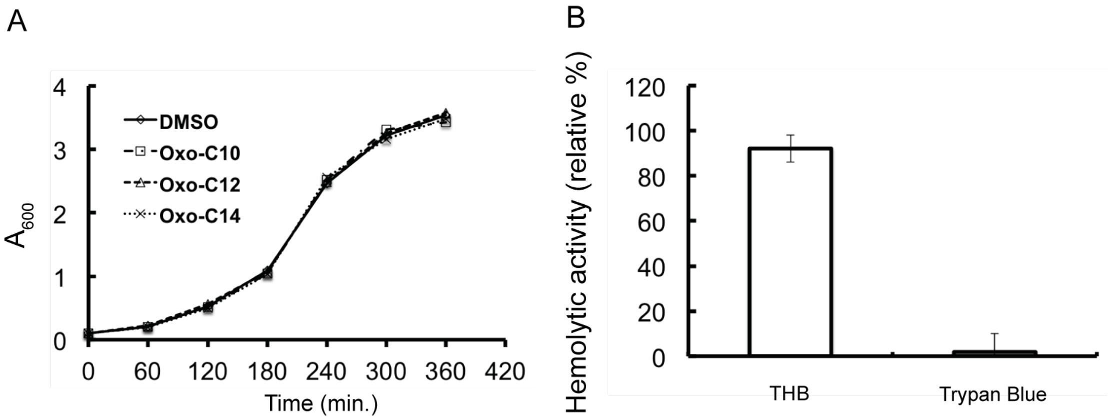

Supplementary Figure S2. Bactericidal effect of oxo-C12-HSL on *S. pyogenes* M1

Effect of glucose and iron on the *S. pyogenes* M1 growth inhibition by oxo-C12-HSL was examined.

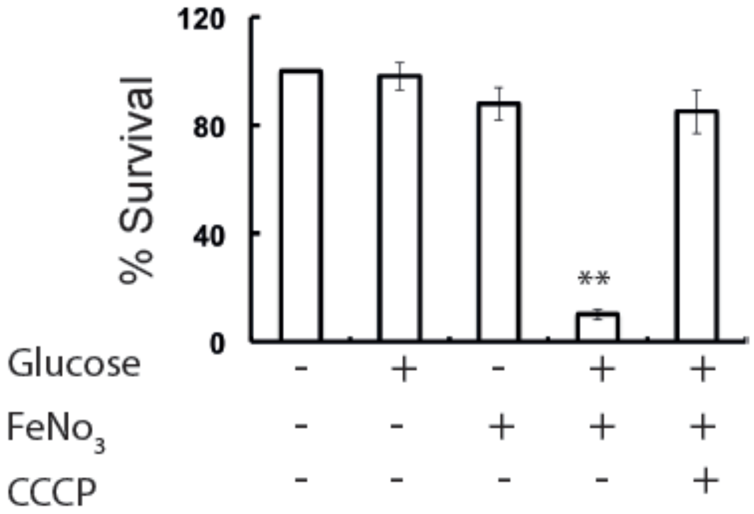

Supplementary Figure S3. Growth Curve of *S. pyogenes* M6 S165 in CM prepared from *P. aeruginosa.* The growth *of S. pyogenes* M6 S165 remains unaffected in the CM.

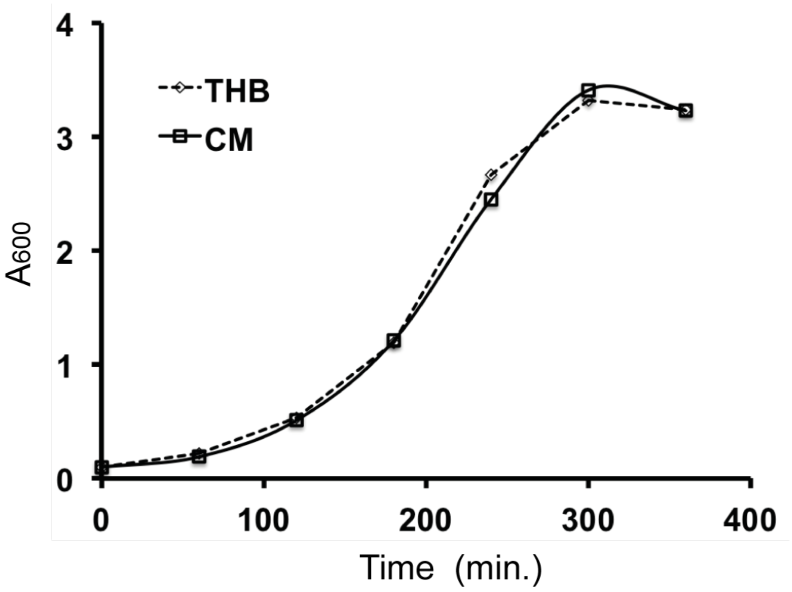

Supplementary Figure S4. Growth curve and hemolytic activity of *S. pyogenes* M6 S165 harboring luxR under gyrA promoter.

(A) Growth curve (B) Hemolytic activity during different stages of growth

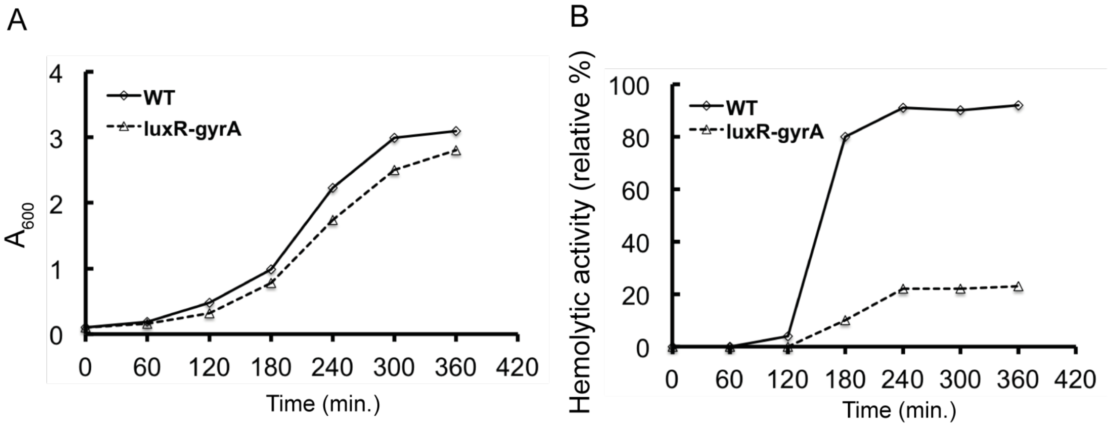

Supplement: Supplementary Data [file srep44902-s1.pdf]
